# Ethical considerations of neuro-oncology trial design in the era of precision medicine

**DOI:** 10.1007/s11060-017-2502-0

**Published:** 2017-05-29

**Authors:** Saksham Gupta, Timothy R. Smith, Marike L. Broekman

**Affiliations:** 1 Department of Neurosurgery, Cushing Neurosurgery Outcomes Center, Brigham and Women’s Hospital, Harvard Medical School, Boston, USA; 2 Department of Neurosurgery, Brain Center Rudolf Magnus, University Medical Center Utrecht, Utrecht, The Netherlands

**Keywords:** Precision medicine, Neuro-oncology, n-of-1 trials, Clinical trial design, Medical ethics

## Abstract

The field of oncology is currently undergoing a paradigm shift. Advances in the understanding of tumor biology and in tumor sequencing technology have contributed to the shift towards precision medicine, the therapeutic framework of targeting the individual oncogenic changes each tumor harbors. The success of precision medicine therapies, such as targeted kinase inhibitors and immunotherapies, in other cancers have motivated studies in brain cancers. The high specificity and cost of these therapies also encourage a shift in clinical trial design away from randomized control trials towards smaller, more exclusive early phase clinical trials. While these new trials advance the clinical application of increasingly precise and individualized therapies, their design brings ethical challenges . We review the pertinent ethical considerations for clinical trials of precision medicine in neuro-oncology and discuss methods to protect patients in this new era of trial design.

## Introduction

Precision medicine has garnered attention in the public sphere since President Barack Obama declared it a national priority as part of a major US public health initiative. This new paradigm builds on the idea that cancers that harbor certain oncogenic mutations may be particularly susceptible to therapies that specifically target the aberrant proteins created by these mutations. As next-generation sequencing techniques have begun to uncover some of the mutations that drive the formation and growth of various cancers, applying this new therapeutic framework has the potential to revolutionize cancer management.

The last decade has seen many innovations in the usage of targeted therapies. Vemurafenib, a targeted kinase inhibitor of the B-Raf protein variant produced by a V600E point mutation in *BRAF*, has been shown to reduce tumor burden and to improve overall survival in melanoma patients [1]. Similarly, gefitinib and erlotinib prolong survival for patients with non-small cell lung cancer by inhibiting overactive epidermal growth factor receptor caused by activating mutations in *EGFR* [2, 3]. Precision medicine potentially has tremendous applicability in neuro-oncology and will allow patients to receive individualized therapies that target the genetic mutations underlying their diseases [4]. Indeed, early phase n-of-1 and small cohort investigational trials studies have already begun to demonstrate the potential of precision medicine in neuro-oncology [5–7].

The successes and potential of precision medicine have also introduced new challenges not only related to clinical care, but also to ethics of clinical research. While there remains little doubt of the potential improvements in efficacy with precision medicine, characterizing and understanding these challenges can uphold the standards of patient autonomy, respect, and protection. In this discussion, we review pertinent ethical challenges to early phase clinical trials in precision medicine.

## Ethical challenges of early phase clinical trials for precision medicine

New trials of targeted therapies are tested in patients whose cancers harbor the specific mutations those therapies target. This design makes recruitment for large-scale randomized clinical trials (RCTs) exceedingly difficult since patients selected for a trial will not only need to share the same cancer and stage, but must share similar oncogenic mutations as well. Clinical trial design will reflect the specificity and expense of targeted therapy as it shifts from large RCTs towards early phase testing in n-of-1 and small cohort trials. For rare brain tumors, recruiting large cohorts of patients with the similar genomic profiles becomes nearly impossible, so early phase trials circumvent the challenge of relatively low prevalence of brain cancers but introduce new ethical challenges that must be addressed.

Advances in NGS have made these problems especially relevant as they have provided individual patients and their providers the ability to assess the genomic composition of their tumors to optimize their management [8]. These technologies compare the genetic composition of tumor and matched germline tissues to identify the tumorigenic mutations each patient’s cancer harbors. The Dana Farber Cancer Institute and other centers currently screen all resected brain tumors with NGS technology for specific oncogenic variations to identify patients who may benefit from an approved targeted therapy or qualify for an investigational trial [9]. These data may be used to efficiently select therapeutics for further study on a larger scale and also, to develop databases that help guide decision-making [10]. Selected agents must also demonstrate the ability to penetrate the blood brain barrier and reach a therapeutic level in preclinical experiments to render neuro-oncologic trials scientifically valid.

We focus this discussion on the most salient ethical challenges to early phase clinical trials in neuro-oncology, including informed consent, societal value, generalizability, institutional oversight, vulnerable patient selection, and justice. These ethical issues are relevant considerations in other types of trials, though the costs, specificity, and prerequisite of NGS of precision medicine trials raise novel concerns that are compounded by the relatively low prevalence of brain cancers and potential cognitive changes.

### Informed consent

Physicians must secure informed consent before sequencing patients’ tumors for entry into trials of targeted therapy. Informed consent requires physicians to explain details about the benefits and risks of a procedure or therapy accurately and understandably to patients. The rapidly changing landscape of genomic medicine requires physicians to understand the theory of precision medicine and be familiar with various therapies in order to inform patients of their options. Physicians differ in their levels of knowledge and experience with precision medicine, which may preclude the ability to provide adequate informed consent [11]. Even for physicians with knowledge on the topic, the limited health literacy of patients combined with complex medical jargon can opacify treatment options [12, 13]. The complexity of genomics in neuro-oncology and targeted therapy mechanisms may fortify the communication barriers that already exist between physicians and patients [8]. Furthermore, NGS exposes patients to discovering incidental genetic mutations not relevant to their entry into a trial. The content and format of informed consent must be adapted to overcome these challenges.

Informed consent requests must incorporate options for communicating incidental findings. In the clinical trial context, NGS will be used to screen for specific oncogenes that determine entry into a trial. It may also reveal the presence of incidental mutations in the same or different genes [14]. The outcry in response to the American College of Medical Genetics’ since rescinded recommendations to broaden screening for genomic variants in newborns without permission demonstrates the importance of respecting the right not to know about genetic mutations [15]. This same ethical principle applies in oncology, in which patients’ rights to not know about incidental mutations in their germline or tumor samples.

The four types of incidental findings in NGS are genetic variants that are medically actionable for the patient, medically actionable for the patient’s family but not the patient, associated with disease but not currently medically actionable, and of unknown significance [16]. Non-disclosure of incidental findings that are medically actionable may prevent interventions that would improve a patient’s outcomes health. Additionally, the discovery of incidental findings in this context can reveal germline mutations that lead to identification and management of family members at risk for cancer [17]. Consequently, NGS findings hold ethically salient consequences for family members who may tangentially learn medically relevant facts about their own genome that they did not consent to know. This phenomenon also affects the privacy of enrollees’ biological family members since the enrollee may learn about health risks in other family members by learning about their own genome. These findings may distress family members and subsequently affect familial social relationships in addition to potentially breaching the rights to not know and to privacy.

The disclosure of gene variants that accurately predict the development of diseases that cannot be prevented or managed may cause patient psychological distress, but allow them to adjust their life plans around this illness. Gene variants of unknown medical significance are also possible outcomes of NGS. Their disclosure likely carries no utility but may be stored in a medical record in case its significance becomes uncovered. Developing NGS technologies that only probe for mutations of interest for each individual trial circumvents the discovery of incidental findings altogether, but may be financially prohibitive and impedes the discovery of medically actionable variants. Each of these four potential scenarios must be clearly delineated in the informed consent process, and oversight committees should consider how to inform patients about the different types of incidental findings and how to create opt-in or opt-out systems for their disclosure.

The format in which informed consent is obtained must efficaciously communicate detailed descriptions of oncogenes of interest, targeted therapies, and potential incidental findings. The process must also be concise enough for patients to complete it without confusion or fatigue from the complex knowledge and different options they must weigh. Multidisciplinary teams can help design resources for patients and physicians to aid in this process; for example, genetic counselors may aid oncologists in communicating complex genetic information and implementing flexible consent processes [18]. Tiered informed consent approaches involve multiple stages of obtaining consent and provide patients the time to understand one element of the trial at a time [19]. The most central and necessary information about a trial is presented first, while less significant and more complex details like incidental findings are presented at an appropriate later time. Patients often have difficulty understanding and responding to the presence of mutations with unknown clinical significance, so any new informed consent structure must also include adequate educational resources to explain the uncertainty around these [20].

### Societal value

Each individual targeted therapy requires significant financial investment for research and development and is applicable to a narrow spectrum of patients, limiting its societal value [21]. An argument could be made that cheaper behavioral interventions that reduce the burden of oncological disease, such as smoking cessation, improved diet, and exercise, should be funded instead of more expensive targeted therapies. However, oncology should not be an “either-or” enterprise. As public health initiatives combat risk factors like cigarette smoking and obesity, the average lifespan continues to increase and age is an unmodifiable risk factor for many cancers. Also, many brain cancers, including medulloblastoma, glioblastoma, and atypical meningioma, do not have strongly associated modifiable risk factors. Even for those that do, the avoidance of risk factors does not guarantee the prevention of cancer. In addition, certain NGS technologies are becoming less expensive than routine MRIs, and smaller trials are less expensive to organize than RCTs.

There are also reasons to believe that despite the high up-front costs of a targeted precision therapy, it provides societal benefit beyond its immediate application as a therapy for a specific cancer. One such example is “drug repositioning”, the concept that drugs designed to target an aberrant protein in one cancer can be applied to other cancers with the same molecular disruption. For example, study of the V600E mutation in *BRAF* in melanoma led to the development of B-Raf inhibitors that years later, have shown potential efficacy in craniopharyngioma [5]. Early phase targeted therapy trials also contribute towards the general understanding of the significance of various mutations as potential oncogenes, which advances scientific knowledge in general. In any case, the majority of Americans support precision medicine initiatives [22].

### Generalizability

RCTs often enroll dozens or hundreds of patients, providing high enough enrollment to allow for detailed statistical analysis of the effects of the new treatment. Smaller cohorts often have limited generalizability as the confidence interval for any measure of an outcome varies inversely with cohort size. In practical terms, a clinician determining whether to prescribe a new targeted therapy to a patient based on the results of a trial with two enrollees may be unable to determine whether their patient will respond similarly to those enrollees. If those enrollees also responded differently to the therapy despite having the same mutational profile, the clinician cannot accurately predict which enrollee the patient would respond more similarly to. Brain pathologies also face the unique challenge that therapeutics must cross the blood brain barrier.

The goal of precision medicine is to provide patient-specific therapies that maximize outcomes, including minimizing toxicity. However, even if multiple small trials show no evidence of toxicity for patients receiving a specific therapy, physicians cannot accurately determine potential toxicities without evidence from large and longitudinal cohort studies [23]. As with any therapeutic regimen characterized by uncertain efficacy and toxicity, this lack of knowledge should be communicated to patients. While a full understanding of the potential harms of a treatment that undergoes trials cannot be comprehensively known, physicians may weigh its potential benefits against proxy approximations of risk, such as the toxicity associated with other therapies that have the same pharmacological target or by extrapolation of a therapy’s toxicity profile in other cancers.

### Oversight

Institutional oversight is a requirement for clinical research. In the United States, any research that uses human test subjects requires Institutional Review Board (IRB) approval. The role of the IRB includes determining if there is enough preclinical evidence to justify a trial in humans.

The specificity of targeted therapies could increase the difficulty of accumulating sufficient preclinical evidence to justify any trial. In vivo testing would require the development of animal models of cancers that match the specificity of all study populations’ genomic signatures, which may be unfeasible. New experimentation protocols and emerging technologies such as ex vivo organoids and analytical data modeling can mitigate this problem, but ultimately, oversight committees must set strict and consistent evidential standards to warrant approval. Additionally, oversight committees have a role in ensuring that the therapeutics that were developed for other cancers and tested in neuro-oncology trials achieve therapeutic levels in the brain by crossing the blood brain barrier without increasing risk of systemic toxicity. Testing therapies that have not been proven to achieve therapeutic levels in the brain would bring further ethical challenges related to patient safety.

Last, the proliferation of early phase trials threatens to bring less oversight; it is much more manageable for an oversight committee to follow a large trial than many small trials, especially as the distinction between research and individualized care becomes less clear. Oversight committees will need to adapt to these anticipated changes to ensure appropriate adjustments that maintain sufficient oversight. While the function of these committees will remain the same, their structure should reflect the increasing number and complexity of these trials.

### Vulnerable patient selection

Neuro-oncologic patients enrolling in trials require protections given vulnerabilities inherent to brain disease and to potentially having refractory cancers. Brain pathologies and their therapies may subtly alter a patient’s decision-making capacity. The poor prognoses of many brain cancers may incentivize patients to explore experimental treatments. These problems are exacerbated for late-stage patients and those with refractory disease, such that these vulnerable patients may become the most eager group to join trials. This is especially problematic for Phase 1 trials, but also has implications for trials with therapeutic intent, which constitute the majority of precision medicine trials.

Equitable participant selection seeks to prevent the exploitation of vulnerable patients in clinical trials. Even though studies are being designed to test targeted treatments upfront, experimental targeted therapies are also tested in patients whose cancers remain refractory to traditional therapeutic modalities. Even though the primary goal of many precision medicine trials is related to care, at the same time, most investigators also have the goal to generate knowledge to improve management of future patients with similar disease profile as their enrollees. This implies that trial investigators should not continually seek the most desperate patients as enrollees only to then prioritize prescribing the tested therapy to healthier eligible patients. Ensuring that the characteristics of trial enrollees reflect their intended beneficiaries becomes more difficult to accomplish in small cohorts, so care must be taken to carefully define enrollee criteria and generalizability of a study.

### Justice

The selection criteria for early phase targeted therapy trials will include specific genetic data that necessitate access to genetic sequencing technologies and the expertise to interpret genetic data. Patients of low socioeconomic status may not have equal access to these resources that are often located in advanced tertiary care institutions [8]. Consequently, both the enrollees and beneficiaries of these trials are more likely to be patients of high socioeconomic status, thereby excluding patients of low socioeconomic status. Furthermore, patients of different socioeconomic statuses face differential carcinogenic exposures, such as higher rates of cigarette smoking in poorer patients, which can lead to differential oncogenic changes.

It is important to note that just recruitment is a challenge in all clinical trials. The requirement of NGS technologies in addition to standard studies like MRI brings an additional impediment for patients of lower socioeconomic status. Therapies specific for genetic mutations that are more common in patients of low socioeconomic status due to certain environmental exposures may therefore never be developed, which would propagate these patients’ exclusion from precision medicine. Therefore, patients from lower socioeconomic backgrounds require increased protection to ensure their access to and representation within these trials [24, 25].

Genomic data gathered for these trials might also risk unfair treatment of enrollees by societal actors. The Genetic Information Nondiscrimination Act enacted in 2008 provides broad prohibitions for health care insurance and employer from discriminating based on known genetic predispositions for future disease. It is not all-encompassing; for example, insurers may request genetic data to determine whether to cover certain procedures [26]. Despite these protections, many patients and even some physicians fear potential discrimination and do not know the rights they have over their genetic data [26, 27]. These fears may be augmented by H.R. 1313, a piece of legislation recently introduced in Congress that exempts employer wellness programs from “limitations under the Genetic Information Nondiscrimination Act of 2008 on collecting the genetic information of employees or family members of employees” [28]. Protections against genetic discrimination should remain to maintain just societal treatment for patients and their families and to maintain their privacy.

## Conclusions and future directions

There has been a surge in early phase trials of precision medicine that target mutations specific to individual tumors. These therapies hold immense potential to improve outcomes in neuro-oncology, but it is necessary to consider the implications of the changes associated with these trials, including their high costs and small cohort sizes, to protect patients and uphold scientific standards. Ethical considerations for clinical trials of precision therapies include informed consent, vulnerable patient protection, societal value, generalizability, institutional oversight, and justice. While each of these present problems for early phase trials of targeted therapies, trial design can adapt to ensure ethical standards are met.

Obtaining informed consent provides major challenges towards these trials due to the difficulty of communicating information and the handling of incidental findings. Innovative informed consent structures such as tiered and multi-disciplinary approaches to educate patients will aid in this process [25]. In a recent cohort of cancer patients who underwent tumor sequencing with an NGS technology, 1% of reports revealed incidental findings [17]. This prevalence will vary according to the NGS methodology applied and cancer being studied; nevertheless, informed consent processes must include options for the patient to determine how to handle such scenarios. Many centers currently include these scenarios in informed consent processes, though no consensus has been achieved on the optimal way to accurately convey this information. Patient surveys on the clearest information and language to communicate these scenarios will aid in the development of standardized methods to achieve informed consent.

While long-term societal value of an innovation is difficult to predict, the clinical and scientific benefits from targeted therapies should be recorded and analyzed to determine their true cost-effectiveness. This can be done by unbiased third-party institutions with the expertise to conduct economic analyses in biomedical fields. Societal value also depends on researchers’ clearly defining the generalizability of each trial as they become smaller and more specific, including specific mutations and demographic details. Adjustments in trial end-points and statistical methodology will aid in achieving higher societal value and more precise generalizability. End-points may include biomarker values, radiographic change, and symptomatic control in additional to traditional end-points like 1 year progression-free survival. Small sample size statistics such as Bayesian probabilistic modelling may also aid in analyzing results [29].

The composition of oversight committees will need to reflect the diversity of the geneticists, oncologists, and biologists who contribute to the development of targeted therapies. Patients and their representatives may also provide input to determine the priorities and agenda of the development of precision medicine to increase public trust and involvement and improve equitability [30, 31].

Likewise, trial participants themselves should be representative of society and should not be limited to only those who can afford access. Patient and community advocacy groups may develop infrastructure to help patients of low SES gain access to these trials, whether by easing the burdens of physically accessing tertiary centers, enrolling patients in affordable insurance plans, and so on. The integration of genomic results into patients’ medical records must come with protections against genetic discrimination by employers, insurers, and other institutions [32]. Ethicists who analyze the social and professional underpinnings of this increasingly common form of discrimination need to coordinate with clinical investigators and policymakers closely to ensure such protections are in place.

These trials have specific, ethically pertinent impacts on the families of trial enrollees. The potential for an enrollee’s NGS to uncover findings that impact family members directly impinges on family members’ autonomy and privacy. An opt-in system, whereby family members could affirm their desire to learn of clinically relevant inheritable variants they are at risk for, would protect autonomy but may require burdensome coordination by the patient. Family members that do not or cannot complete this request (estranged relatives, children, people who lack capacity, etc) could be assumed to have opted in to be informed of the potential that they harbor medically actionable variants as most people desire to ascertain information that could improve or elongate their lives [33]. Knowledge of latent oncogenic drivers of brain cancer may become an issue of justice for family members, as timely diagnosis may impact prognosis. On the contrary, enrollees’ own right to privacy allows them to determine which personal data may become known to family members regardless of the impact it may have on their family’s health.

Novel trial design paradigms may mitigate many of the ethical challenges. For instance, large-scale multi-institutional studies increase sample size, thereby increasing the societal value and generalizability of a study. The larger patient pool also allows for enrollment of demographically and socioeconomically diverse cohorts, thereby addressing concerns for justice. An example is the ACT IV study, a double-blind RCT of rindopepimut for EGFRvIII*-*positive glioblastoma with currently over 200 centers participating. The ACT IV study has been a successful model of a multicenter RCT of a precision medicine in neuro-oncology, but is not without challenges. As broader networks of trial investigators are necessary for these types of trials, this will raise novel ethical issues, including limitations to generalizability and societal value from inter-institutional inconsistencies in patient selection, treatment, imaging, and pathologic analysis. In addition, this trial structure complicates the coordination, standardization, and implementation of institutional and central oversight.

Other emerging clinical trial designs that might mitigate some of the ethical challenges include blinded single participant “on–off” studies, increased enrollment from community centers, seamless phase II/III studies, and Bayesian adaptive randomization models amongst other solutions that also bring their own new ethical challenges [34]. Combinatorial approaches will also be applied in early phase trials and may be scaled up like the ACT IV study, potentially bringing the ethical challenges associated with both types of trials. Novel approaches may address ethical concerns related to the scientific validity, societal value, oversight, and generalizability of the study, but do not directly address the concepts of informed consent, vulnerable patient protection, and justice. Patient advocacy groups consisting of patients and their families may aid in simplifying the informed consent process and maintaining just treatment of patients. These groups may also collaborate with physicians to ensure vulnerable patient protection and to determine the priorities that matter most to patients in situations wherein different ethical principles clash.

While these are largely beyond the scope of this analysis, NGS and precision medicine raise challenging ethical questions regarding information sharing and management after the completion of trials. Incidental findings with unknown clinical significance discovered by NGS in these trials may potentially be studied to determine their role in health and disease. The prospective usage of incidental findings should ideally be assessed prior to enrollment and included in informed consent requests. Retrospective usage of such data should also require informed consent to protect patients from discrimination and respect their privacy.

We acknowledge the meritorious scientific background of early phase trials: decades of molecular biology and genomics research settings have yielded findings that have dramatically elongated patient survival and improved quality of life for cancer patients. Increasingly precise therapies have the potential to benefit neuro-oncologic patients immensely. However, as oncological management transitions to increased specificity and personalization, ethical analysis of these changes and adaptation of researchers and IRBs to new standards are imperative to protect patients.
